# PSATF-6mA: an integrated learning fusion feature-encoded DNA-6 mA methylcytosine modification site recognition model based on attentional mechanisms

**DOI:** 10.3389/fgene.2024.1498884

**Published:** 2024-11-12

**Authors:** Yanmei Kang, Hongyuan Wang, Yubo Qin, Guanlin Liu, Yi Yu, Yongjian Zhang

**Affiliations:** ^1^ School of Cyber Science and Engineering, University of International Relations, Beijing, China; ^2^ College of Computer Science and Technology, Guangdong University of Technology, Guangzhou, China

**Keywords:** N6-methylcytosine (6 mA), integrated learning, transfer learning, cross -species, DNA methylation N6-methylcytosine (6 mA), DNA methylation

## Abstract

DNA methylation is of crucial importance for biological genetic expression, such as biological cell differentiation and cellular tumours. The identification of DNA-6mA sites using traditional biological experimental methods requires more cumbersome steps and a large amount of time. The advent of neural network technology has facilitated the identification of 6 mA sites on cross-species DNA with enhanced efficacy. Nevertheless, the majority of contemporary neural network models for identifying 6 mA sites prioritize the design of the identification model, with comparatively limited research conducted on the statistically significant DNA sequence itself. Consequently, this paper will focus on the statistical strategy of DNA double-stranded features, utilising the multi-head self-attention mechanism in neural networks applied to DNA position probabilistic relationships. Furthermore, a new recognition model, PSATF-6 mA, will be constructed by continually adjusting the attentional tendency of feature fusion through an integrated learning framework. The experimental results, obtained through cross-validation with cross-species data, demonstrate that the PSATF-6 mA model outperforms the baseline model. The in-Matthews correlation coefficient (MCC) for the cross-species dataset of rice and m. musus genomes can reach a score of 0.982. The present model is expected to assist biologists in more accurately identifying 6 mA locus and in formulating new testable biological hypotheses.

## 1 Introduction

DNA methylation is a common epigenetic phenomenon, whereby methyl groups are attached to the bases of a DNA molecule through covalent bonds, thus affecting gene expression and cellular functions. Among these, 6-methyladenine (the sixth position of the purine ring in adenine, 6 mA) is one of the most significant epigenetic modifications in the DNA molecule (He et al., 2019), which plays a pivotal role in biological functions and pathological processes, such as cell differentiation, development, cell cycle regulation, chromosome stability, and cellular tumourigenesis (Guo et al., 2020). In order to further investigate the effects of methylation at the 6 mA site on DNA, a number of more mature biological experimental techniques have been developed (McIntyre et al., 2019). These include single-molecule real-time (SMRT) sequencing methods based on high-throughput technologies (Flusberg et al., 2010), as well as liquid chromatography-tandem mass spectrometry (LC-MS/MS) (Greer et al., 2015), laser-induced fluorescence capillary electrophoresis (CE-LIF) (Krais et al., 2010) and immunoprecipitation (Pomraning et al., 2009). However, these traditional bioanalytical techniques are highly laborious and time-consuming for the identification of 6 mA sites, and are unable to perform accurate methylation localisation in large-scale sequences (Zhang et al., 2021).

With the continuous development of machine learning and deep learning, some researchers have already investigated tool models for identifying 6 mA locus based on machine learning methods (Kong and Zhang, 2019), but they still fall short of the efficiency and accuracy of cross-species identification. Liu and Li, (2020) constructed the SICD6mA and SNNRice6mA models using recurrent neural networks, enabling the identification of 6 mA locus on an independent rice genome with an accuracy of over 90%. Furthermore Huang et al. (2021), integrated a long-term and short-term memory artificial neural network with an attention mechanism to enhance the recognition accuracy. The previously described method was successful in identifying the 6 mA site, but it employed a single DNA coding method, which overlooked the chemical nature of the nucleotides and the relationship between the relevant nucleotides. Additionally, several other notable methods have emerged Tang et al. (2022). Proposed Deep6mAPred, employed a parallel stacking of CNNs and Bi - LSTMs along with an attention mechanism, enhanced the prediction accuracy. Subsequently Tsukiyama et al. (2022), combined the BERT model with vectors to incorporate the nucleotide relationships at different positions into the model, which further enhanced the prediction ability Zheng et al., (2023). Adopted adaptive embedding for nucleotide encoding and combined multi - scale CNNs and long short - term memory for feature extraction, achieving favorable accuracies Huang et al. (2023). Improved stacking ensemble model for predicting DNA N6 - methyladenine site, utilized a meta - learning algorithm with cross - validation output as input to the final classifier and exhibited excellent performance in the Rosaceae independent test.

Despite the availability of a range of models at home and abroad, the recognition methods all face challenges in terms of their single encoding method and poor generalisation and cross-species recognition abilities. In this paper, we propose a new encoding method based on the advantages of deep learning technology in feature learning and neural network technology. Our method employs position-specific encoding of DNA sequences to join the automatic attention mechanism of neural networks for optimisation. This enables the identification of key features of the 6 mA site by better capturing the probability distribution of the target site in the sequence. Once the existing machine learning and deep learning models have been validated, it can be demonstrated that the proposed encoding method fully considers the positional frequency of the sequence, and has a superior ability to predict the 6 mA locus.

## 2 Materials and methods

### 2.1 Literature search strategy

We utilise the DNA 6 mA benchmark dataset, which is a combination of the rice genome (Chen et al., 2019), the mouse muscle genome, and a cross-species dataset that is a mixture of these two benchmark datasets. In order to reduce sequence redundancy in the dataset, the threshold was set to 0.8 using CD-HIT software in this paper (Fu et al., 2012).

The rice genome benchmark dataset comprises 1760 sequences, of which 880 are designated as positive samples and 880 as negative samples. The benchmark dataset for the rat muscle genome has 3,868 sequences, of which 1,934 are positive samples and 1,934 negative samples. The cross-species dataset comprises 5,484 sequences, of which 2,768 are positive samples and 2,716 are negative samples. In all benchmark datasets, the length of each sequence is 41 nt. Details of the dataset can be found in Table 1.

**TABLE 1:** Summary of dataset.

Species	Dataset	Number of Samples
Corss-species	Positive	2,768
	Negative	2,716
Rice	Positive	880
	Negative	880
M.musculus	Positive	1,934
	Negative	1,934

**FIGURE 1:**
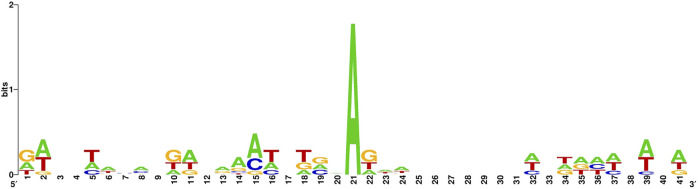
6 mA site sample in the benchmark datase.

The sequence identity of the 6 mA site sample in the benchmark dataset is illustrated in Figure 1.

### 2.2 Feature encoding methods

#### 2.2.1 Binary encoding

The binary coding method employs a four-bit binary vector to represent each nucleotide in a DNA fragment. For example, the nucleotide A is represented by the binary vector (1,0,0,0), the nucleotide C by (0,1,0,0), the nucleotide G by (0,0,1,0), and the nucleotide T by (0,0,0,1). This binary coding scheme is also one of the most basic coding schemes in biogenetics (Feng et al., 2019), with the advantage of being able to accurately describe the different positions of the ribonucleotides in the same sample of sequences (Liu et al., 2019).

#### 2.2.2 K-nucleotide frequency coding (Kmer)

Kmer frequency coding refers to the division of a nucleotide sequence under study into strings containing k bases. In general, a nucleotide sequence of length m can be divided into (m-k + 1) kmers (Nie and Zhao, 2019). This paper investigates and analyses the problem of multi-nucleotide sequences with k consecutive nucleotides in order to accomplish k-nucleotide frequency coding. As an illustration, if k = 3, there are 4^3^ = 64 dinucleotide frequencies to be calculated, such as AAA, AAT, AAG, AAC, etc. The remaining nucleotides are TTT. It can be observed that the k-mer is employed to elucidate the relationship between bases in a nucleotide sequence through the application of statistical frequencies derived from the original nucleotide sequence. This approach is used to elucidate the relationship between bases in a nucleotide sequence.

The calculation formula for k-mer is presented in Equation 1.

$$f\left(t\right)=\frac{N\left(t\right)}{N},t\in \left\{\text{AAA},\text{AAC},.\text{AAG},..\text{TTT}\right\}$$ (1)

#### 2.2.3 Nucleotide chemical property codes (NCP)

Nucleotide Chemical Property Coding is a feature extraction method that aims to extract the intrinsic information between nucleotides. There are four different types of nucleotides in the DNA sequence, each of which has a different chemical structure and binding properties. All types of nucleotides can be classified into three main categories based on their chemical properties. In this paper, the four types of nucleotides ACGT are coded as (1,1,1), (0,1,0), (1,0,0), (0,0,1) based on their chemical properties.

#### 2.2.4 Enhanced nucleotide composition (ENAC)

The Enhanced Nucleotide Composition (ENC) coding scheme is based on calculating nucleotide composition (NAC) based on a fixed-length window of sequences (Liu F et al., 2018; Chen et al., 2018a; Chen et al., 2018b). This will use a fixed-length window (in this paper, the window size is set to 5), starting from the start position of each nucleotide sequence and sliding along the sequence, to compute the composition of the nucleotides within the window is calculated. The dimensionality of the ENAC coding is determined by two parameters, including the sequence length and sliding window size. The latter can be calculated as (sequence length - window size + 1)*4. The calculation formula is shown by Equation 2:

$$f\left(t\right)=\frac{N\left(t\right)}{N},t\in \left\{A,C,G,T\right\}$$ (2)

In this formula, the $N\left(t\right)$ numerator represents the number of nucleotides, while the denominator N represents the length of the ribonucleotide sequence.

#### 2.2.5 Position-specific trinucleotide statistical coding (PSTNPds)

The PSTNPds encoding employs a statistical strategy based on DNA double-strand features (Chen et al., 2020), which results in a more distinctive statistical profile based on complementary base pairing (Li et al., 2023). In this context, A and T are considered to be identical to C and G. Consequently, for each sample, it can be converted to a sequence containing only A and C. This process results in the generation of 2³ = 8 trinucleotides: AAA, AAC, Thus, for a DNA sequence of length L, its trinucleotide position specificity can be represented by a matrix of 8*(L-2).

$$\begin{array}{c} Z=\left[\begin{array}{cccc} z_{1,1} & z_{1,2} & \cdots & z_{1,L-2}\\ z_{2,1} & z_{2,2} & \cdots & z_{2,L-2}\\ \vdots & \vdots & \vdots & \vdots \\ z_{8,1} & z_{8,2} & \cdots & z_{8,L-2} \end{array}\right]\\ z_{i,j}=F^{+}\left(3m_{er}|j\right)-F^{-}\left(3mer_{i}|j\right)i=1,2,\cdots ,8,j=1,2,\cdots ,L-2 \end{array}$$ (3)

In Equation 3, the symbol “ $F^{+}\left(3mer_{i}|j\right)$ and $F^{-}\left(3{\text{mer}}_{i}|j\right)$ ” represents the frequency of the I th trinucleotide at the j th position in the positive and negative data set.

### 2.3 PSATF-6mA model

The PSATF-6mA model proposed in this paper is a fusion of neighbouring nucleotide position encoding with multi-head neural network self-attention. This generates a weight matrix for the 6 mA sites in the DNA sequence, which is used to identify the relevant positions. Ultimately, this generates the main framework of the model through the integration of the learning network, which is used to continuously optimise the feature matrix. The model framework is described as follows.

**FIGURE 2:**
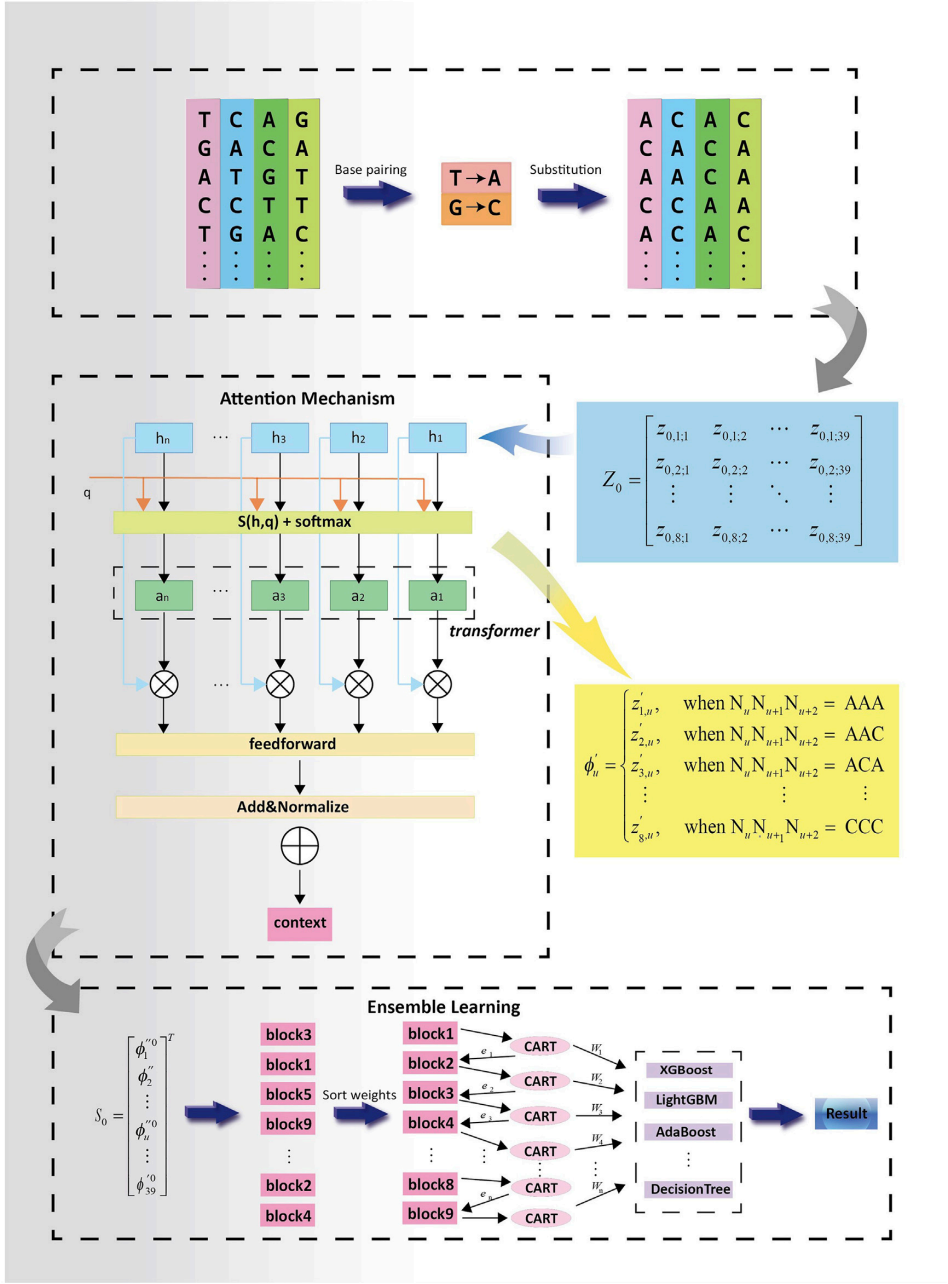
Experimental flowchart of PSATF-6mA model.

The PSATF-6mA model primarily encodes the original DNA sequence with the neighbour nucleotide positional features, thereby fully considering the positional relationship between neighbouring nucleotides. Furthermore, the position fusion feature matrix is incorporated into the attention mechanism via a feed-forward neural network. This enables the statistical characteristics of the position probability relationship formed by base complementary pairing to be fully exploited after encoding, thereby greatly improving the accuracy and learnability of the encoding as the input vector for machine learning. In the neural network model, the multi-head attention mechanism is employed to assign higher-quality weights to the nucleotides that are more pertinent to the identification of the 6 mA site. Furthermore, the feed-forward neural network is utilised to continuously optimise the feature vectors following the completion of the encoding process. Subsequently, the model is evaluated using the cross-entropy loss function. A series of decision tree models is generated through iterative training of the decision tree models based on residuals. The final integrated result is obtained by weighted averaging the predictions of each decision tree. The final prediction results are then obtained. The overall design concept of the PSATF-6mA model proposed in this paper is illustrated in Figure 2.

The DNA sequence was initially converted to a sequence comprising adenine (A) and cytosine (C) utilising a coding process to transform the DNA sequence. It is crucial to highlight that each of the trinucleotide combinations in the position-specific trinucleotide statistical coding (AAA, AAC, ACA, etc.) are paired according to complementary bases, thereby exhibiting a more distinctive statistical signature. At this juncture in the paper, A and T are considered to be identical to C and G, and thus, for each sample, it can be converted to a sequence containing only A and C. Consequently, there are a total of 2³ = 8 trinucleotide compositions: AAA, AAC, CCC. For a sample in the dataset with a DNA length of 41 bp, the details of the trinucleotide position specificity can be represented by the following 8 *39 matrix.

$$Z_{0}=\left[\begin{array}{cccc} z_{0,1;1} & z_{0,1;2} & \cdots & z_{0,1;39}\\ z_{0,2;1} & z_{0,2;2} & \cdots & z_{0,2;39}\\ \vdots & \vdots & \ddots & \vdots \\ z_{0,8;1} & z_{0,8;2} & \cdots & z_{0,8;39} \end{array}\right]$$

The model is querying the coding representation of the variant construction samples. In this instance, the sample $S_{0}$ is represented by the first feature, which $\left(\phi_{0}\right)$ corresponds to a specific trinucleotide fragment. Consequently, the sample comprises the aforementioned components, where the latter is defined as follows:

$$\phi_{u}^{\prime }=\left\{\begin{array}{ccc} z_{1,u}^{\prime }, & \text{ when }N_{u}N_{u+1}N_{u+2}=\text{AAA } & \\ z_{2,u}^{\prime }, & \text{ when }N_{u}N_{u+1}N_{u+2}=\text{AAC } & \\ z_{3,u}^{\prime }, & \text{ when }N_{u}N_{u+1}N_{u+2}=\text{ACA } & \\ \vdots & \vdots & \vdots \\ z_{8,u}^{\prime }, & \text{ when }N_{u}N_{u+1}N_{u+2}=\text{CCC } & \end{array}\right.$$

In the event that the trinucleotide fragment at a given position is AAA, the result is $\phi_{0}^{u}=z_{0}^{2},u$ . Conversely, if the trinucleotide fragment at a position is AAC, the result is $\phi_{0}^{u}=z_{0}^{2},u$ . Consequently, the trinucleotide fragment at the final position, CCC, can be expressed as $\phi_{0}^{u}=z_{0}^{8},u$ . Subsequently, the coding $z_{0}$ representation of sample $S_{0}$ was constructed based on the frequency of the trinucleotide fragment. Finally, this paper combines all $\phi_{0}^{u}$ elements into a single vector, designated as $S_{0}$ (where h denotes horizontal stacking and T denotes the transpose operation).

$$S_{0}={\left[\begin{array}{c} \phi_{1}^{\prime \prime 0}\\ \phi_{2}^{\prime \prime }\\ \vdots \\ \phi_{u}^{\prime \prime 0}\\ \vdots \\ \phi_{39}^{\prime 0} \end{array}\right]}^{T}$$

The additive attention mechanism invoked in the PSATF-6mA model is designed to handle the case where the vector dimensions of the Query and Key do not coincide. This is achieved through the following Equation 4:

$$a\left(q,k\right)=W_{v}^{T}\tanh\left(W_{q}q+W_{k}k\right)$$ (4)

$q\in R^{q},k\in R^{k},W_{q}\in R^{h\times q},W_{k}\in R^{h\times k},W_{v}\in R^{h\times v}$ . The Query and Key can be unified into a single vector dimension through the integration of two fully connected layers. This enables the relationship between the query and key to be determined by summing and activating the tanh function. Subsequently, the vector dimension can be unified with that of the value through the integration of a fully connected layer $W_{v}$ . The final result is then passed through the function to $softmax\left(W_{v}^{T}\tanh\left(W_{q}q+W_{k}k\right)\right)$ obtain the probability distribution of the query and key, which is used to adjust the attention weight. The dot product scoring function is frequently employed as an attention mechanism, as it is capable of capturing the similarity between vectors. Furthermore, the features of DNA sequences are typically represented as vectors or matrices. The dot product scoring function is capable of measuring the correlation between a query and a key, which may result in its effectiveness in identifying positional features in some sequence-based tasks.

Subsequently, a cross-entropy loss function is employed based on the actual 6 mA bit labels, and the positional frequency feature matrix is trained and optimised. The cross-entropy loss function is employed to quantify the discrepancy between the probability distribution of the model output and the actual labels in a block. Furthermore, the gradient statistics of the position frequencies are sorted from the highest to the lowest. Subsequently, several classification decision trees are constructed to rectify the residuals generated in the preceding step, until the results of all the decision trees are integrated to complete the final prediction. In the design of applying the multi-head attention mechanism to the PSATF-6mA model, the key is to query a specific pattern or key information corresponding to the attention to the 6 mA locus on the DNA sequence. By dynamically adjusting the query, the model is able to focus on different DNA segments in different contexts, thereby improving its adaptability with regard to the encoding process.

The addition of the attention mechanism allows for the formation of keys and values that correspond to specific trinucleotide fragments in the DNA sequence and their corresponding statistical features. By considering the similarity of the keys and values, it is possible to capture more precise information from the DNA sequence. The use of an additive attention mechanism as a scoring function in PSTNPds facilitates the calculation of similarity scores between different trinucleotide fragments. The selection of an appropriate scoring function facilitates the measurement of relatedness in DNA sequences, thereby enabling the attention mechanism to identify important fragments with greater accuracy.

### 2.4 Attention mechanism in PSATF-6mA

In the PSATF-6mA, the attention mechanism plays a pivotal role in the identification of DNA - 6 mA methylcytosine modification sites. It enables the model to automatically focus on the information that is more crucial for recognizing 6 mA sites during the processing of DNA sequences. Specifically, for different encoding schemes such as Kmer, NCP, ENAC, and particularly the PSTNPds emphasized in this study, the attention mechanism assigns an attention weight to each encoded feature. These weights reflect the degree of attention that the model pays to each feature during the decision-making process.

**FIGURE 3:**
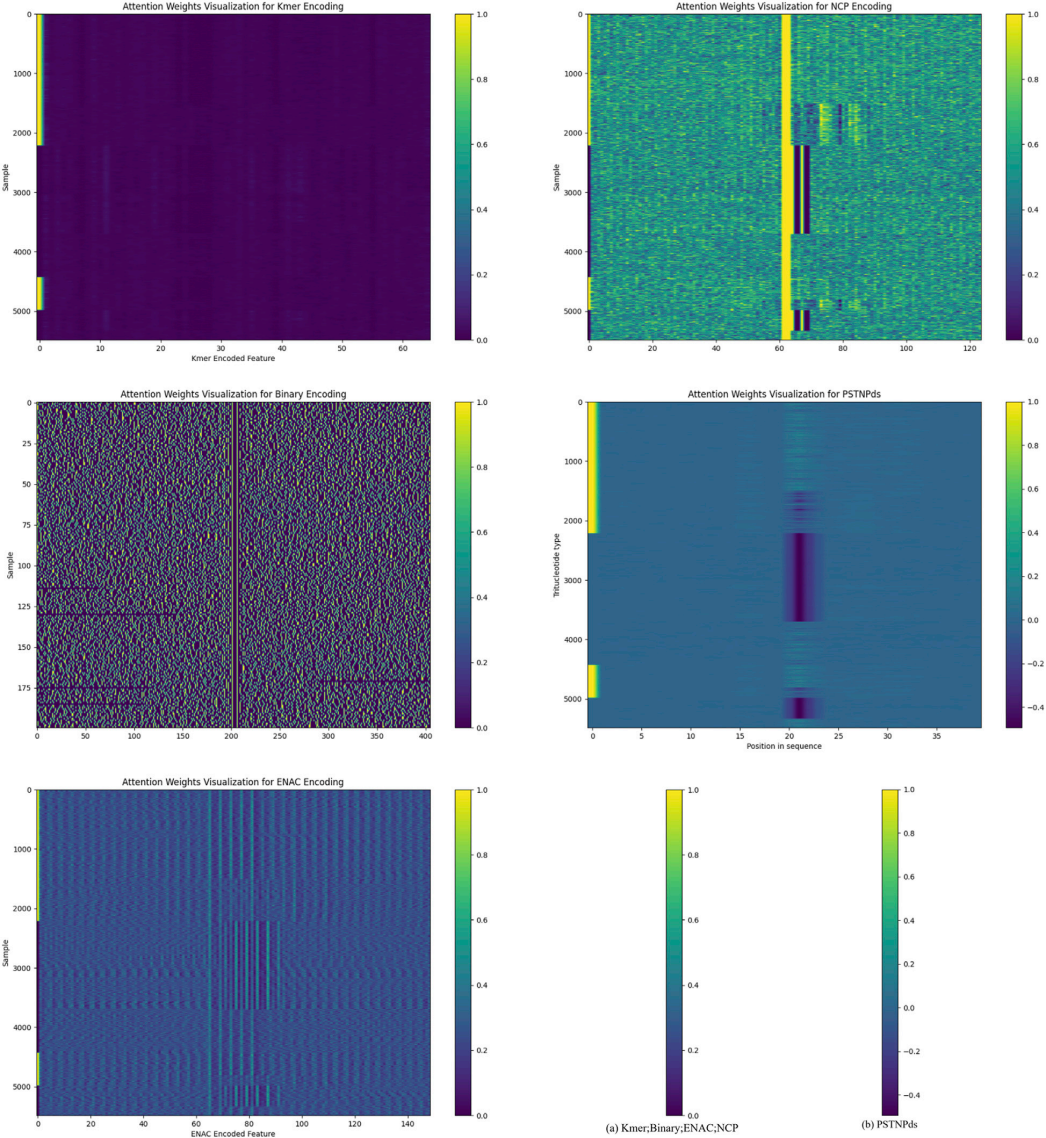
Interpretation of the attention mechanism through heat maps.

We demonstrate the attention mechanism of the model in Figure 3. The attention heat maps provide a visual representation of the attention weights. Overall, if certain regions in the heat map exhibit a generally darker color, it indicates that the model assigns a higher attention weight to the features corresponding to these regions. This implies that these features are regarded as more important sources of information when the model determines the presence of 6 mA sites.

For different encoding methods, the *x*-axis of the heat map corresponds to different encoded features. For instance, in the kmer-encoded heat map, each tick on the *x*-axis represents a specific kmer combination. If the area corresponding to a particular kmer combination on the *x*-axis shows a darker color in the heat map, it suggests that the model pays higher attention to this kmer combination during data processing. This could be because this kmer combination has a certain association with the occurrence of 6 mA sites in the DNA sequence, and the model has learned to capture this association and assigns corresponding attention during the decision-making process.

Similarly, for the NCP encoding, the features on the *x*-axis correspond to different nucleotide chemical property categories or their combinations. The darker regions indicate that the model pays more attention to the features related to these chemical properties, suggesting a close relationship between these chemical properties and the recognition of 6 mA sites. On the *y*-axis, each tick represents a sample. By observing the color distribution of different samples in the heat map, it can be seen that the model’s attention to different samples also varies. Some samples may have a darker color throughout the map, indicating that the model pays a higher overall attention to all the features of these samples. This may be because these samples contain more information related to 6 mA sites or their feature patterns are more easily recognizable by the model. In contrast, for some samples with a lighter color, the model may consider the information they contain to be of relatively lower importance for determining 6 mA sites.

The attention mechanism enables PSATF-6mA to utilize the input encoded feature information more effectively. Instead of treating all features equally, it can dynamically adjust the focus according to the relevance between the features and the target (6 mA site identification). This allows the model to better capture critical information when dealing with complex DNA sequence data, thereby improving the model’s accuracy and performance in identifying 6 mA sites.

## 3 Results

### 3.1 Model validation

In this paper, the following metrics were employed for the assessment of sensitivity (Sn), specificity (SP), accuracy (ACC) and Mathews correlation coefficient (MCC) (Yu et al., 2020a; Yu et al., 2020b; Liu et al., 2015; Jiang et al., 2019; Wang et al., 2016):

$$\begin{array}{c} Pre=\frac{TP}{FP+TP}\\ SN=\frac{TP}{TP+FN}\\ SP=\frac{TN}{FP+TN}\\ ACC=\frac{TP+TN}{TP+TN+FP+FN}\\ MCC=\frac{TP\times TN-FP\times FN}{\sqrt{\left(TP+FN\right)\left(TP+FP\right)\left(TN+FP\right)\left(TN+FN\right)}} \end{array}$$

Where TP and TN are the number of true positive and true negative samples, and FP and FN are the number of false positive and false negative samples, respectively. Additionally, this paper employs subject operating characteristic (ROC) curves (Liu and Chen, 2020; Wang et al., 2021; Liu and Li, 2020) and precision-recall (PR) (Liu and Chen, 2020; Yu et al., 2020a) curves to provide a visual representation of the prediction performance. The ROC curves are generated by plotting the true-positive rate (*y*-axis) and the false-positive rate (*x*-axis) at varying thresholds. The closer the ROC curve is to the upper left corner, the better the performance (Tang et al., 2022). The PR curve is a plot of precision (*y*-axis) *versus* recall (*x*-axis) at various thresholds. Recall is equivalent to Sn, The area under the ROC curve and the PR curve (AUC) are employed to quantify the prediction efficacy (Chen et al., 2020; Chen et al., 2019; Li et al., 2021; Liu et al., 2016). The AUC is defined as a value between 0 and 1, with one representing optimal prediction, 0.5 indicating random prediction, and 0 representing the opposite of a prediction. A larger AUC value indicates a more accurate prediction.

### 3.2 Comparison experiments of different coding approaches

In order to compare the effect of the PSATF-6mA model based on the enhancement of encoding methods by using a multi-head attention mechanism in the task of DNA-6mA methyl cytosine modification site recognition, a comparison will be made between the following methods: Four commonly used DNA encoding methods, namely, Kmer, NCP, ENAC, and Binary, were selected for the experiment. The integration learning module in PSATF-6mA model selected LightGBM (Pal and Mitra, 1992; Meng, 2017), XGboost, Adaboost (Freund and Schapire, 1995), and DecisionTree, respectively, as the integration learning classifiers used in this paper. The K-fold cross-validation method is an effective model evaluation method. This paper uses the 5-fold cross-validation method to evaluate the effectiveness of the prediction model (Shi et al., 2019; Khanal et al., 2021; Xie et al., 2020; Buzhong et al., 2019).

Table 2 presents the results of a comparative analysis between the PSATF-6mA model and the traditional coding method Kmer. The PSATF-6mA model, which employs LightGBM and DecisionTree as integrated learning classifiers, demonstrated superior coding effectiveness compared to Kmer. The model performance of Sn was enhanced by 45.9%. The results demonstrate that the PSATF-6mA model, when integrated with LightGBM and DecisionTree, outperforms the traditional coding method Kmer. The model’s performance in terms of Sn, ACC, and MCC is significantly enhanced, with improvements of 45.9, 29.984, and 0.7758, respectively. Furthermore, the ROC metric also shows a notable improvement, with an increase of 0.3663.

**TABLE 2:** The PSATF-6mA model selects LightGBM and DecisionTree as integrated classifiers.

Model	Method	Sn	Sp	Pre	Acc	MCC	AUROC	AUPRC
Light GBM	binary	75.3920	74.3180	78.9040	74.8540	0.4981	0.7771	0.7953
	Kmer	53.5040	66.2360	62.3560	59.8100	0.2011	0.6325	0.6549
	NCP	75.3920	74.3180	78.9040	74.8540	0.4981	0.7771	0.7953
	ENAC	67.0460	73.1020	74.9700	70.0380	0.4068	0.7504	0.7811
	**PSATF-6mA**	**99.4580**	**98.1960**	**98.2920**	**98.8360**	**0.9769**	**0.9988**	**0.9988**
Decision Tree	binary	67.8480	65.8460	69.0860	66.8480	0.3387	0.6685	0.7658
	Kmer	62.1760	58.8780	60.8180	60.5400	0.2108	0.6053	0.7104
	NCP	66.9460	64.9620	68.4320	65.9560	0.3212	0.6595	0.7603
	ENAC	65.9680	62.8280	66.2420	64.4060	0.2881	0.6440	0.7470
	**PSATF-6mA**	**92.1600**	**93.2280**	**93.3620**	**92.6880**	**0.8543**	**0.9274**	**0.9270**

The bold font represents the experimental results of the model in this article.

Table 3 lists the performance of the test on XGBoost and Adaboost classifiers. The PSATF-6mA model performs better with the selection of XGBoost and Adaboost as integrated learning classifiers. The model’s Sn improves. The results demonstrate that the PSATF-6mA model outperforms the baseline model in terms of accuracy, with improvements of 11.78%, 23.01%, and 20.5352% for precision, recall, and F1-score, respectively. Furthermore, the model exhibits enhanced Matthews correlation coefficient (MCC) values of 0.4175 and 0.4318, indicating a more balanced performance. Additionally, the receiver operating characteristic (ROC) curve demonstrates an improvement of 0.1573 and 0.1739, indicating a more accurate classification.

**TABLE 3:** The PSATF-6mA model selects XGBoost and Adaboost as integrated classifiers.

Model	Method	Sn	Sp	Pre	Acc	MCC	AUROC	AUPRC
XGBoost	binary	87.7140	69.6840	79.3940	78.7740	0.5651	0.8418	0.8493
	Kmer	63.0420	75.1920	74.3940	69.0540	0.3929	0.7505	0.7722
	NCP	87.2440	70.4560	79.5340	78.9180	0.5725	0.8229	0.8250
	ENAC	80.5980	72.4060	79.4540	76.5320	0.5296	0.8172	0.8313
	**PSATF-6mA**	**99.4940**	**98.7500**	**98.8060**	**99.1260**	**0.9826**	**0.9991**	**0.9991**
AdaBoost	binary	74.3880	73.9860	78.7220	74.1780	0.4914	0.8125	0.8236
	Kmer	65.3160	68.7860	69.0540	67.0320	0.3438	0.7268	0.7307
	NCP	74.3880	74.0600	78.7260	74.2160	0.4922	0.8123	0.8233
	ENAC	76.0460	71.6680	76.980	73.8660	0.4813	0.8094	0.8107
	**PSATF-6mA**	**97.3980**	**94.7760**	**95.3220**	**96.1000**	**0.9232**	**0.9864**	**0.9797**

The bold font represents the experimental results of the model in this article.

### 3.3 Comparison experiments with existing models

In order to verify the generality of the PSATF-6mA model for DNA-6mA methylcytosine modification site recognition method on deep learning models, this paper selects three deep learning models, CNN(Muhammad et al., 2019), RNN and ResNet (He et al., 2016), which are currently the most widely used in deep learning. In order to ensure consistency with the evaluation metrics, this paper employs the same metrics, including accuracy, sensitivity, specificity, MCC, and AUC. The experimental results are shown in Table 4. Additionally, the current state-of-the-art recognition models are selected for comparison, with the results of SNNRice6mA and SpineNet-6mA (Yu and Dai, 2019) directly quoted from the literature (Abbas et al., 2020). In all evaluation metrics, the model in this paper outperforms previous models.

**TABLE 4:** Comparative results with traditional deep learning models.

Model	Sn	Sp	Pre	Acc	MCC	AUROC	AUPRC
CNN_model	24.1840	82.6800	84.6800	53.1360	0.1347	0.7768	0.8010
RNN_model	38.5520	81.5020	82.4500	59.8120	0.2688	0.7733	0.7966
ResNet_model	60.6560	60.7980	62.0140	60.7220	0.2150	0.6024	0.5957
PSATF-6mA (Decision Tree)	92.1600	93.2280	93.3620	92.6880	0.8543	0.9270	0.9474
PSATF-6mA (AdaBoost)	97.3980	94.7760	95.3220	96.1000	0.9232	0.9864	0.9797
PSATF-6mA (LightGBM)	99.4580	98.1960	98.2920	98.8360	0.9769	0.9988	0.9988
AttNet (XGBoost)	99.4940	98.7500	98.8060	99.1260	0.9826	0.9991	0.9991

**FIGURE 4:**
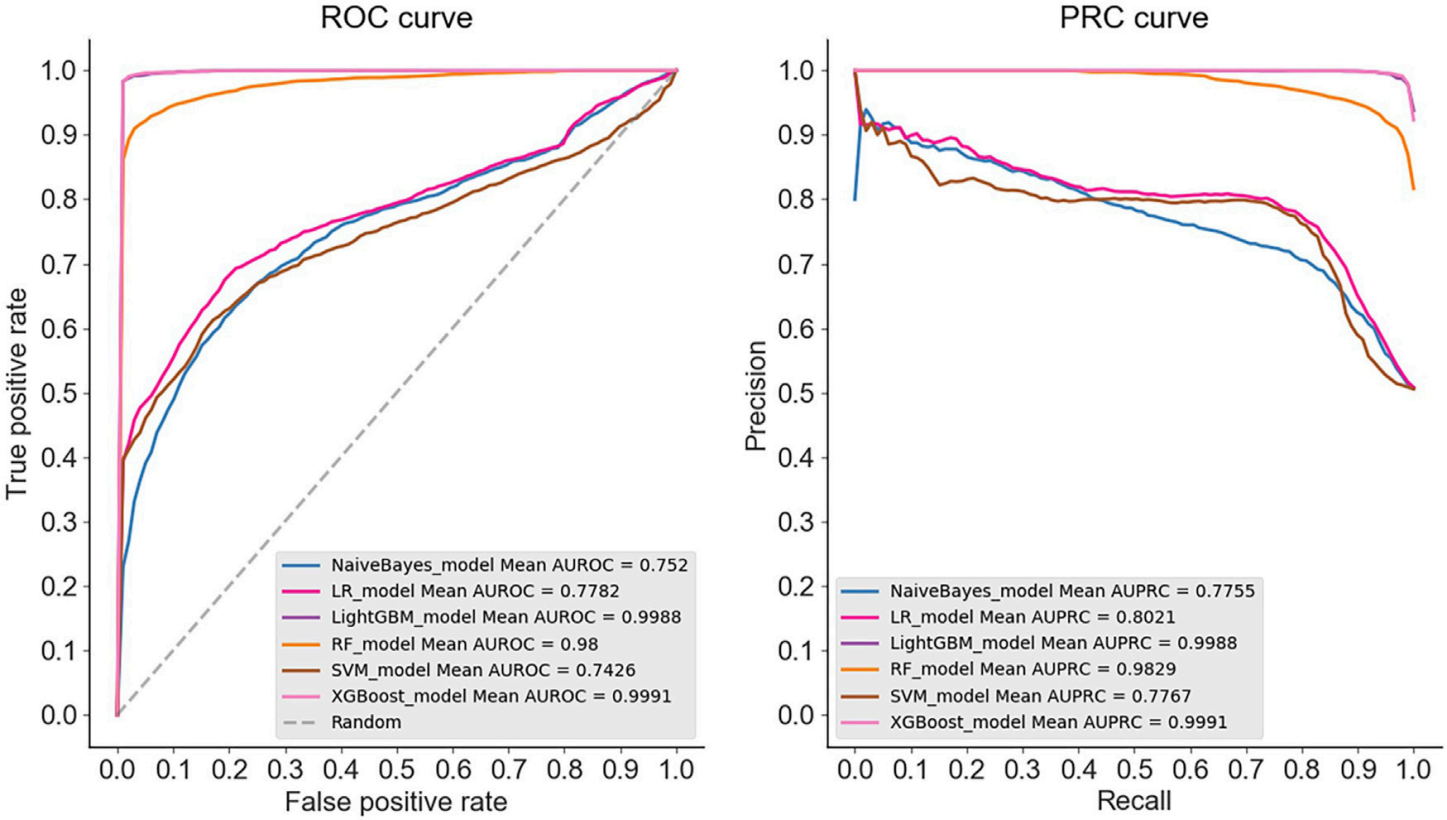
Prediction results between different integrated learning classifiers selected for PSATF-6mA model.

**FIGURE 5:**
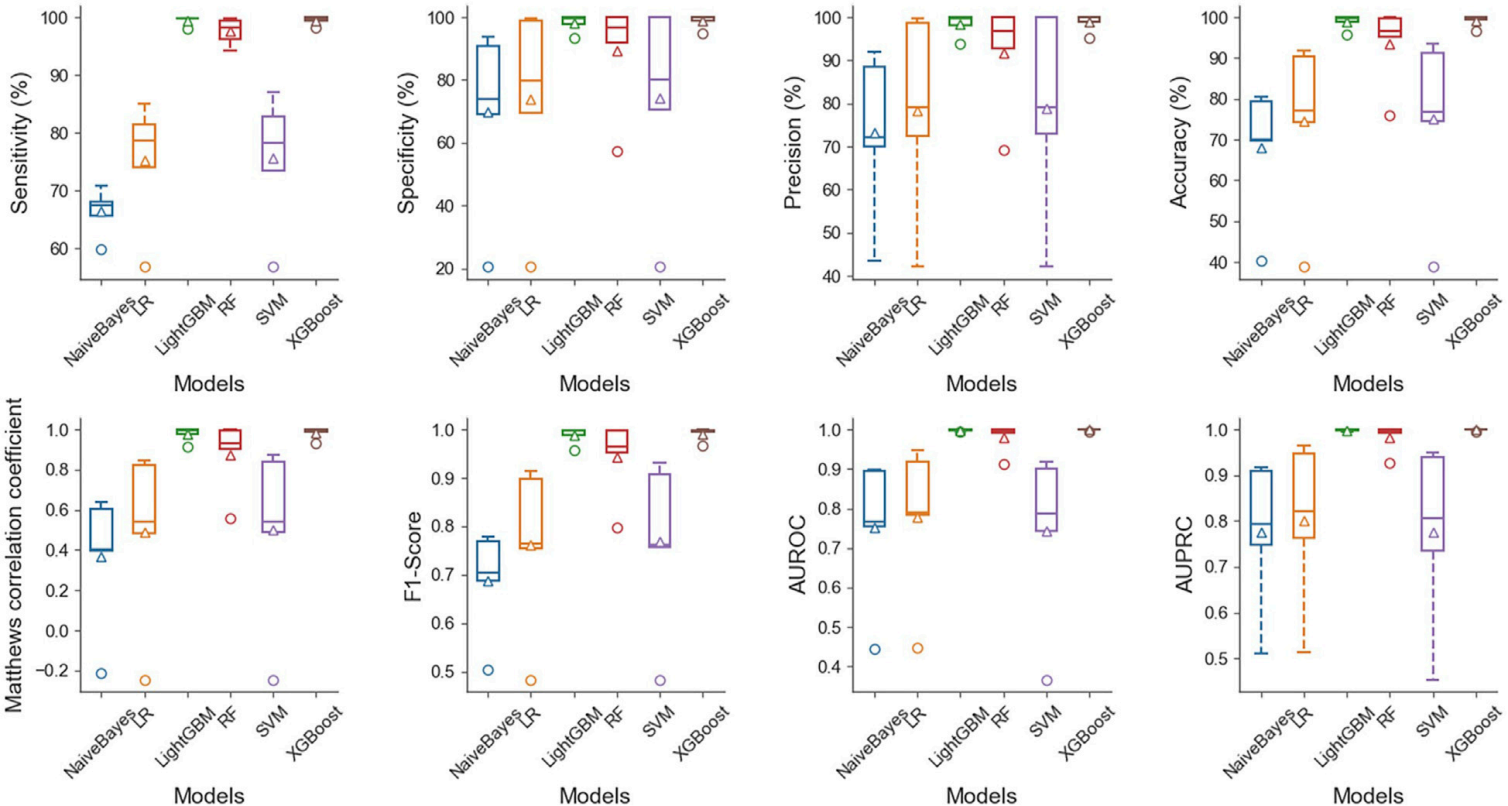
Comparative analysis of evaluation metrics between different integrated learning classifiers selected by PSATF-6mA model.

The results demonstrate that in the case of multiple machine learning and deep learning models as classifiers, the enhancement of using the PSATF-6mA model for the traditional model leads to a more significant improvement in the ACC value compared to existing models, with the exception of the ResNet model. The value of AUPRC can reach up to 0. In the case of the machine learning model, when the PSATF-6mA model integrated learning classifier selects XGBoost, the ACC value of the model can be as high as 99.126, while the ACC value of the integrated learning classifier selects LightGB can be as high as 98.836. The improvement is notable; once more, it demonstrates the efficacy of the PSATF-6mA model in identifying the 6 mA locus. To provide a further visual illustration of the model’s performance, this paper employs model histograms and PRC and ROC curves. The comparison of evaluation metrics for different ensemble learning classifiers in the PSATF-6mA model is shown in Figures 4, 5.

To demonstrate the superiority of the proposed scheme, PSATF-6mA is comprehensively compared with existing predictors, namely, IDNA6MA-PseKNC (Feng et al., 2019), csDMA (Liu et al., 2019), and iIM-CNN (Wahab et al., 2019) with respect to their respective advantages. The results are presented in Table 5, which employs a 5-fold cross-validation approach. The species predictors are evaluated on the same dataset. The sensitivity, specificity, accuracy, Matthews correlation coefficient (MCC), and area under the receiver operating characteristic curve (AUROC) of the model in this paper are improved by 0.125, 0.207, 0.167, 0.3316, and 0.1071, respectively, over the existing state-of-the-art models. This further demonstrates the superiority of the PSATF-6mA model.

**TABLE 5:** Comparative analysis of existing predictors with this model.

Species	Model	Sn	Sp	Acc	MCC	AUROC
Cross_species	iIM-CNN	0.8690	0.7800	0.8240	0.6510	0.8920
	csDMA	0.8630	0.7350	0.7990	0.6030	0.8790
	iDNA6MA-PseKNC	0.7620	0.7690	0.7650	0.5310	0.8440
	PSATF-6mA (XGBoost)	0.9940	0.9870	0.9910	0.9826	0.9991

The PSATF-6mA model offers significant advantages over existing methods, particularly in comparison with ilM-CNN, csDMA, and MultiScale-CNN-4mCPred. It utilizes a novel adjacent nucleotide position encoding combined with a multi-head self-attention mechanism, allowing for a nuanced understanding of inter-nucleotide relationships; in contrast, ilM-CNN relies on one-hot encoding, which may inadequately capture positional dynamics, while csDMA employs pseudo amino acid composition (PseAAC) and k-mer strategies that do not fully exploit nucleotide positioning. Additionally, the self-attention mechanism in PSATF-6mA enables dynamic weighting of nucleotides, enhancing feature identification across diverse contexts, whereas ilM-CNN uses fixed weights in convolutional layers, which may limit sensitivity to significant features, and csDMA lacks the adaptive weighting present in PSATF-6mA. Furthermore, PSATF-6mA’s adaptability to various sequence contexts allows it to effectively manage intricate variations, while ilM-CNN may show rigidity in its response to different DNA sequences, and csDMA may also face limitations in handling varying biological data. In terms of overfitting mitigation, while both PSATF-6mA and MultiScale-CNN-4mCPred incorporate dropout layers, the former’s architecture allows for more sophisticated regularization through attention mechanisms, potentially leading to superior generalization in complex datasets. Finally, empirical results consistently show that PSATF-6mA achieves higher Matthews correlation coefficient (MCC) values during cross-validation, indicating enhanced accuracy and reliability in identifying 6 mA sites compared to ilM-CNN, csDMA, and MultiScale-CNN-4mCPred.

In summary, the PSATF-6mA model distinguishes itself by leveraging advanced encoding techniques, dynamic feature extraction, greater flexibility, effective overfitting mitigation, and superior performance metrics, thereby enhancing its efficacy in analyzing complex biological sequences.

## 4 Conclusion

The accurate identification of 6 mA sites in DNA is of great importance for the elucidation of the function of 6 mA epigenetic modifications. This study develops and implements a method called PSATF-6mA modelling approach. The methodology text focuses on the statistical strategy of DNA double-stranded features, utilising the multi-head self-attention mechanism in neural networks to model DNA position probability relations. Furthermore, the position weights are continuously adjusted through an integrated learning framework. The experimental results demonstrate the merits of the proposed PSATF-6mA model by comparing it with the four most commonly used DNA encoding methods on classifiers.

The superior performance of the PSATF-6mA model for the recognition of 6 mA can be attributed to the following factors. The model demonstrates position sensitivity and sequence correlation. The PSATF-6mA model considers the information of each position in the sequence, as well as the relationship between different parts of the sequence. This is achieved through the use of an attentional mechanism, which takes into account the nucleotides in the sequence that are more relevant to the 6 mA locus. Position sensitivity and sequence correlation help to better represent important features and patterns in the DNA sequence.

The PSATF-6mA model offers several advantages in the context of binary classification problems, such as identifying the 6 mA locus. It employs a transformer’s multi-head attention mechanism, which focuses on the DNA double-stranded features of the statistical strategy. Additionally, it prioritises the computational features of the DNA strand itself. Consequently, it can be used to select a more comprehensive and accurate integrated learning classifier through the integrated learning framework, which facilitates the integration of bioinformatics and computer mathematics. Although the PSATF-6mA model demonstrated satisfactory performance, there are still potential limitations. For instance, the model is not yet sufficiently robust for complex multi-locus knowledge across species. To further enhance the prediction of 6 mA locus, this paper proposes two avenues for future research: (1) The design of a processing scheme for larger cross-species datasets; and (2) the construction of a feature optimisation tool for the features of the DNA sequences themselves, so that the model can better remove the noise from the features during encoding. While there is still room for optimisation of the PSATF-6mA model, this paper believes that it will be utilised as a useful tool to accelerate progress in the detection and understanding of DNA site function.

## Data Availability

Publicly available datasets were analyzed in this study. This data can be found here: http://lab.malab.cn/acy/PTM_data/data/DNA/N6-methyladenine/5%20iIM-CNN%20Data.7z.

## References

[B1] Abbas Z. Tayara H. Chong K. T. (2020). SpineNet-6mA: a novel deep learning tool for predicting DNA N6-methyladenine sites in genomes. IEEE Access 8, 201450–201457. 10.1109/access.2020.3036090

[B2] Buzhong Z. Jinyan L. Lijun Q. Chen Y. Lü Q. (2019). Sequence-based prediction of protein-protein interaction sites by simplified long short-term memory network. Neurocomputing 357 (10), 86–100. 10.1016/j.neucom.2019.05.013

[B3] Chen W. Lv H. Nie F. Lin H. (2019). i6mA-Pred: identifying DNA N6-methyladenine sites in the rice genome. Bioinforma. Oxf. Engl. 35 (16), 2796–2800. 10.1093/bioinformatics/btz015 30624619

[B4] Chen Z. Zhao P. Li F. Leier A. Marquez-Lago T. T. Wang Y. (2018a). *iFeature*: a Python package and web server for features extraction and selection from protein and peptide sequences. Bioinformatics 34 (14), 2499–2502. 10.1093/bioinformatics/bty140 29528364 PMC6658705

[B5] Chen Z He N Huang Y (2018b). Integration of a deep learning classifier with a random forest approach for predicting malonylation sites. Bioinformatics (6), 451–459. 10.1016/j.gpb.2018.08.004 30639696 PMC6411950

[B6] Chen Z. Zhao P. Li F. Wang Y. Smith A. I. Webb G. I. (2020). Comprehensive review and assessment of computational methods for predicting RNA post-transcriptional modification sites from RNA sequences. Briefings Bioinforma. 21 (5), 1676–1696. 10.1093/bib/bbz112 31714956

[B7] Feng P. Yang H. Ding H. Lin H. Chen W. Chou K. C. (2019). iDNA6mA-PseKNC: identifying DNA N6-methyladenosine sites by incorporating nucleotide physicochemical properties into PseKNC. Genomics 111 (1), 96–102. 10.1016/j.ygeno.2018.01.005 29360500

[B8] Flusberg B. A. Webster D. R. Lee J. H. Travers K. J. Olivares E. C. Clark T. A. (2010). Direct detection of DNA methylation during single-molecule, real-time sequencing. Nat. Methods 7 (6), 461–465. 10.1038/nmeth.1459 20453866 PMC2879396

[B9] Freund Y. Schapire R. E. (1997). A depiction-theoretic generalization of on-line learning and an application to boosting. J. Comput. Syst. Sci. 55, 23–37. 10.1007/3-540-59119-2_166

[B10] Fu L. Niu B. Zhu Z. Wu S. Li W. (2012). CD-HIT: accelerated for clustering the next-generation sequencing data. Bioinforma. Oxf. Engl. 28 (23), 3150–3152. 10.1093/bioinformatics/bts565 PMC3516142 23060610

[B11] Greer E. L. Blanco M. A. Gu L. Sendinc E. Liu J. Aristizábal-Corrales D. (2015). DNA methylation on N6-adenine in *C. elegans* . Cell 161 (4), 868–878. 10.1016/j.cell.2015.04.005 25936839 PMC4427530

[B12] Guo Y. Pei Y. Li K. Cui W. Zhang D. (2020). DNA N6-methyladenine modification in hypertension. Aging 12 (7), 6276–6291. 10.18632/aging.103023 32283543 PMC7185115

[B13] He K. Zhang X. Ren S. Sun J. (2016). “Deep residual learning for image recognition,” in 2016 IEEE Conference on Computer Vision and Pattern Recognition (CVPR). Las Vegas, NV, USA, 27-30 June 2016, (IEEE). 10.1109/CVPR.2016.90

[B14] He L. Li H. Wu A. Peng Y. Shu G. Yin G. (2019). Functions of N6-methyladenosine and its role in cancer. Mol. Cancer 18 (1), 176. 10.1186/s12943-019-1109-9 31801551 PMC6892141

[B15] Huang G. Huang X. Luo W. (2023). 6mA-StackingCV: an improved stacking ensemble model for predicting DNA N6-methyladenine site. BioData Min. 16 (1), 34. 10.1186/s13040-023-00348-8 38012796 PMC10680251

[B16] Huang Q. Zhou W. Guo F. Xu L. Zhang L. (2021). 6mA-Pred: identifying DNA N6-methyladenine sites based on deep learning. PeerJ 9 (4), e10813. 10.7717/peerj.10813 33604189 PMC7866889

[B17] Jiang L. Wang C. Tang J. Guo F. (2019). Correction to: LightCpG: a multi-view CpG sites detection on single-cell whole genome sequence data. BMC Genomics 20 (1), 365. 10.1186/s12864-019-5742-x 31084602 PMC6513517

[B18] Khanal J. Tayara H. Zou Q. Chong K. T. (2021). Identifying DNA N4-methylcytosine sites in the Rosaceae genome with a deep learning model relying on distributed feature representation. Comput. Struct. Biotechnol. J. 19 (1), 1612–1619. 10.1016/j.csbj.2021.03.015 33868598 PMC8042287

[B19] Kong L. Zhang L. (2019). i6mA-DNCP: computational identification of DNA N6-methyladenine sites in the rice genome using optimized dinucleotide-based features. Genes 10 (10), 828. 10.3390/genes10100828 31635172 PMC6826501

[B20] Krais A. M. Cornelius M. G. Schmeiser H. H. (2010). Genomic N(6)-methyladenine determination by MEKC with LIF. Electrophoresis 31 (21), 3548–3551. 10.1002/elps.201000357 20925053

[B21] Li F. Liu S. Li K. Zhang Y. Duan M. Yao Z. (2023). EpiTEAmDNA: sequence feature representation via transfer learning and ensemble learning for identifying multiple DNA epigenetic modification types across species. Comput. Biol. Med. 160, 107030. 10.1016/j.compbiomed.2023.107030 37196456

[B22] Li J. He S. Guo F. Zou Q. (2021). HSM6AP: a high-precision predictor for the *Homo sapiens* N6-methyladenosine (m^6 A) based on multiple weights and feature stitching. RNA Biol. 18 (11), 1882–1892. 10.1080/15476286.2021.1875180 33446014 PMC8583144

[B23] Liu F. Lan X. T. Hou T. Kang B. Liu Y. Liu C. (2018). Metagenomic clustering method based on k-mer frequency optimization. Jilin Daxue Xuebao (Gongxueban)/Journal Jilin Univ. Eng. Technol. Ed. 48 (5), 1593–1599. 10.13229/j.cnki.jdxbgxb20170668

[B24] Liu K. Chen W. (2020). iMRM: a platform for simultaneously identifying multiple kinds of RNA modifications. Bioinforma. Oxf. Engl. 36 (11), 3336–3342. 10.1093/bioinformatics/btaa155 32134472

[B25] Liu W. Li H. (2020). SICD6mA: identifying 6mA sites using deep memory network. 10.1101/2020.02.02.930776

[B26] Liu Z. Dong W. Jiang W. He Z. (2019). csDMA: an improved bioinformatics tool for identifying DNA 6 mA modifications via Chou’s 5-step rule. Sci. Rep. 9 (1), 13109. 10.1038/s41598-019-49430-4 31511570 PMC6739324

[B27] Liu Z. Xiao X. Qiu W. R. Chou K. C. (2015). iDNA-Methyl: identifying DNA methylation sites via pseudo trinucleotide composition. Anal. Biochem. 474, 69–77. 10.1016/j.ab.2014.12.009 25596338

[B28] Liu Z. Xiao X. Yu D. J. Jia J. Qiu W. R. Chou K. C. (2016). pRNAm-PC: predicting N(6)-methyladenosine sites in RNA sequences via physical-chemical properties. Anal. Biochem. 497, 60–67. 10.1016/j.ab.2015.12.017 26748145

[B29] McIntyre A. B. R. Alexander N. Grigorev K. Bezdan D. Sichtig H. Chiu C. Y. (2019). Single-molecule sequencing detection of N6-methyladenine in microbial reference materials. Nat. Commun. 10 (1), 579. 10.1038/s41467-019-08289-9 30718479 PMC6362088

[B30] Meng Q. (2017). “LightGBM: a highly efficient gradient boosting decision tree,” in Proceedings of the 31st Conference on Neural Information Processing Systems (NIPS 2017). Long Beach, CA, USA: Curran Associates Inc.

[B31] Muhammad T. Hilal T. To C. K. (2019). iDNA6mA (5-step rule): identification of DNA N6-methyladenine sites in the rice genome by intelligent computational model via Chou’s 5-step rule. Chemom. Intelligent Laboratory Syst. 189 (2019), 96–101. 10.1016/j.chemolab.2019.04.007

[B32] Nie Z. Zhao Y. (2019) “The research of BP neural network based on ant colony algorithm in port throughput prediction,” in 2019 11th international conference on measuring technology and mechatronics automation (ICMTMA). IEEE. 10.1109/ICMTMA.2019.00114

[B33] Pal S. K. Mitra S. (1992). Multilayer perceptron, fuzzy sets, and classification. IEEE Trans. neural Netw. 3 (5), 683–697. 10.1109/72.159058 18276468

[B34] Pomraning K. R. Smith K. M. Freitag M. (2009). Genome-wide high throughput analysis of DNA methylation in eukaryotes. Methods (San Diego, Calif.) 47 (3), 142–150. 10.1016/j.ymeth.2008.09.022 18950712

[B35] Shi H. Liu S. Chen J. Li X. Ma Q. Yu B. (2019). Predicting drug-target interactions using Lasso with random forest based on evolutionary information and chemical structure. Genomics 111 (6), 1839–1852. 10.1016/j.ygeno.2018.12.007 30550813

[B36] Tang X. Zheng P. Li X. Wu H. Wei D. Q. Liu Y. (2022). Deep6mAPred: a CNN and Bi-LSTM-based deep learning method for predicting DNA N6-methyladenosine sites across plant species. Methods (San Diego, Calif.) 204, 142–150. 10.1016/j.ymeth.2022.04.011 35477057

[B37] Tsukiyama S. Hasan M. M. Deng H. W. Kurata H. (2022). BERT6mA: prediction of DNA N6-methyladenine site using deep learning-based approaches. Briefings Bioinforma. 23 (2), bbac053. 10.1093/bib/bbac053 PMC8921755 35225328

[B38] Wahab A. Ali S. D. Tayara H. To Chong K. (2019). iIM-CNN: intelligent identifier of 6mA sites on different species by using convolution neural network. IEEE Access 7, 178577–178583. 10.1109/ACCESS.2019.2958618

[B39] Wang X. Zhang Y. Yu B. Salhi A. Chen R. Wang L. (2021). Prediction of protein-protein interaction sites through eXtreme gradient boosting with kernel principal component analysis. Comput. Biol. Med. 134, 104516. 10.1016/j.compbiomed.2021.104516 34119922

[B40] Wang Y. Liu T. Xu D. Shi H. Zhang C. Mo Y. Y. (2016). Predicting DNA methylation state of CpG dinucleotide using genome topological features and deep networks. Sci. Rep. 6, 19598. 10.1038/srep19598 26797014 PMC4726425

[B41] Xie Z. Deng X. Shu K. (2020). Prediction of protein-protein interaction sites using convolutional neural network and improved data sets. Int. J. Mol. Sci. 21 (2), 467. 10.3390/ijms21020467 31940793 PMC7013409

[B42] Yu B. Qiu W. Chen C. Ma A. Jiang J. Zhou H. (2020a). SubMito-XGBoost: predicting protein submitochondrial localization by fusing multiple feature information and eXtreme gradient boosting. Bioinforma. Oxf. Engl. 36 (4), 1074–1081. 10.1093/bioinformatics/btz734 31603468

[B43] Yu B Chen C Zhou H (2020b). GTB-PPI: Predict Protein-protein Interactions Based on L1-regularized Logistic Regression and Gradient Tree Boosting. Bioinformatics 18 (5), 582–592. 10.1016/j.gpb.2021.01.001 PMC8377384 33515750

[B44] Yu H. Dai Z. (2019). SNNRice6mA: a deep learning method for predicting DNA N6-methyladenine sites in rice genome. Front. Genet. 10, 1071. 10.3389/fgene.2019.01071 31681441 PMC6797597

[B45] Zhang Q. Zhang Y. Li S. Han Y. Jin S. Gu H. (2021). Accurate prediction of multi-label protein subcellular localization through multi-view feature learning with RBRL classifier. Briefings Bioinforma. 22 (5), bbab012. 10.1093/bib/bbab012 33537726

[B46] Zheng P. Zhang G. Liu Y. Huang G. (2023). MultiScale-CNN-4mCPred: a multi-scale CNN and adaptive embedding-based method for mouse genome DNA N4-methylcytosine prediction. BMC Bioinforma. 24, 21. 10.1186/s12859-023-05135-0 PMC9847203 36653789

