# Supplementary material for: PSATF-6mA: an integrated learning fusion feature-encoded DNA-6 mA methylcytosine modification site recognition model based on attentional mechanisms
Source: Front Genet. 2024 Nov 12;15:1498884. doi: 10.3389/fgene.2024.1498884 (PMC11588721; doi:10.3389/fgene.2024.1498884)
Supplement: Supplementary file 1 [file Presentation1.pptx]

## Slide 1
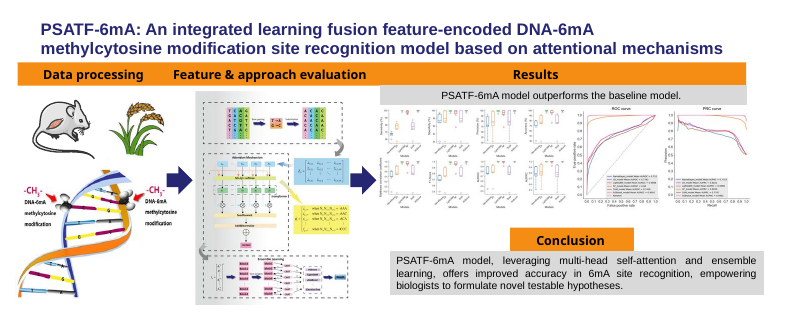

PSATF-6mA: An integrated learning fusion feature-encoded DNA-6mA
methylcytosine modification site recognition model based on attentional mechanisms
 Data processing Feature & approach evaluation Results
PSATF-6mA model outperforms the baseline model.
Conclusion
PSATF-6mA model, leveraging multi-head self-attention and ensemble learning, offers improved accuracy in 6mA site recognition, empowering biologists to formulate novel testable hypotheses.
